# Determination of The Properties of Rat Brain Thermostable Protein Complex which Inhibit Cell Proliferation

**DOI:** 10.22074/cellj.2018.4835

**Published:** 2017-11-04

**Authors:** Diana Dzidziguri, Irina Modebadze, Ekaterine Bakuradze, Giorgi Mosidze, Manana Berulava

**Affiliations:** 1 Department of Biology, Faculty of Exact and Natural Sciences, Iv. Javakhishvili Tbilisi State University, Tbilisi, Georgia; 2 Faculty of Natural Sciences and Healthcare, Sokhumi State University, Tbilisi, Georgia

**Keywords:** Hippocampus, Inhibition of Proliferation, Protein Complex, Tissue Specificity, Transcription

## Abstract

**Objective:**

Cell proliferation is known to be controlled by many networks of regulatory proteins. These multiple and complicated mechanisms of control are still being investigated. The aim of the present study is to determine the different properties of adult rat brain thermostable protein complex (TPC) which affect cell proliferation.

**Materials and Methods:**

This experimental study used brain, kidney and liver tissue from adult (150-170 g) and adolescent (7, 10, 21, 28 days) white rats, adult pigeons and mice. Brain TPC was isolated by alcohol extraction, and primary antibodies Ki67 and GAD65/67 were used for immunohistochemistry, evaluation of transcriptional activity of the tissues and determination of the mitotic index.

**Results:**

The results show that brain TPC from rats reversibly decreases cell proliferation by inhibiting transcription. The evidence suggests that TPC is not species-specific, but expresses tissue specificity with regards to terminally differentiated cells. Rat brain TPC inhibits mitotic activity of the progenitor cells in the dentate gyrus of adolescent rats, and corresponding with this decrease in the mitotic index the number of Ki67 positive cells increases. Simultaneously, the number of GAD65/67-positive cells in the hippocampus decreases by approximately threefold.

**Conclusion:**

These results indicate that rat brain TPC causes the reversible suppression of cell proliferation through the inhibition of transcription. Inhibitory effects of rat brain TPC leads to an increase the number of cells in the cell cycle, in tissues of adolescent rats.

## Introduction

When studying the living cell, it is extremely important to research the functional and structural features of its proteins. The determination of the occurrence of polyfunctional proteins and formation of their complex networks in eukaryotic cells is considered as a great achievement. The formation of these networks is based on, so-called, protein-protein interactions, disruption of which can lead to the development of various diseases including cancers, neurodegenerative diseases, autoimmune diseases, etc. (1-3). Therefore, analysis of these protein-protein interaction networks may provide targets of therapeutic interest. For example, a set of islet cell-specific proteins have been identified and explored using antibodybased proteomics. Colocalization with insulin and glucagon showed that some proteins (DGCR2, GBF1, GPR44, and SerpinB10) were expressed in beta cells. These antibodies were negative in specimens from persons with long-standing type I diabetes (4).

As a result of intensive studies, knowledge in the field of protein complex formation and function has accumulated in recent years. In addition, this type of dynamic interaction between proteins has been studied not only in various species, but also in different types of tissues and cells. Functions of the individual components of the protein complexes are carried through the protein-protein interaction.

Recently we established that the cells from different tissues (kidney, heart, and pancreas) of the adult rat contain the thermostable protein complex (TPC). This complex inhibits the proliferation of homologous cells due to inhibition of the transcription process. The special feature of the complex is the thermostability of its components (5). TPC from the brain tissue of an adult white rat, contains two groups of different proteins, according to column retention time, 5 and 20 minutes respectively (hydrophobic interaction chromatography), as we have described previously (6). It has been shown that the thermostable protein complex from the rat kidney also inhibits transcription in human kidney tumor cells (postoperative material) *in vitro* (7). From these results, we can assume that research on the TPC enables us to learn more about the precise mechanisms of cell proliferation and their management in order to develop therapeutic approaches in the future. It is known that the tissues of adult organisms have differential ability for self-renewal. Moreover, they lose their self-renewal capacity with age. In this regard, brain cells, the loss of which is irreversible, are particularly interesting. The generation of neurons is limited in most areas of the nervous system to certain periods of development. As recently as 10- 15 years ago it was thought that there was no replacement of dead cells after wear or damage to brain tissue, and that this led to the development of a variety of neurological disorders (8-10). However, brain tissue plasticity, realized through, socalled, progenitor cells in two regions of the brain, has been determined in the last few years. These two areas include the sub-ventricular lateral zone and the dentate gyrus of the hippocampus (11). This plasticity is maintained throughout life. The pluripotent stem cells of the brain are able to divide and differentiate, as well as to functionally integrate into tissue (12).

The origin and the renewal of neurons, as well as of any other type of cell, is a regulated process that occurs with both endogenous and exogenous factors. Many different factors regulating neurogenesis have been identified (13). It is shown, for example that neurogenesis is modulated by environmental factors, such as physical activity, stress and learning (14). Neurogenesis in mammals *in vivo* as well as in vitro is induced by the insulin-like growth factor-1 (IGF-1) (15), and neural stem cell proliferation is stimulated by fibroblast growth factor (16). There is less information about the inhibiting endogenous factors involved in neurogenesis. Based on all above, the goal of this study was the determination of tissue and species specificity of adult rat brain TPC and its effect on the transcriptional and proliferative activity of various tissues.

## Materials and Methods

In this experimental study, we performed morphological and biochemical analyses of brain, kidney and liver tissues from white rats, mice and pigeons. The study was approved by a board of Professors from the Biology Department, Faculty of Exact and Natural Sciences, at Iv. Javakhishvili Tbilisi State University, according to the legal and statutory acts extant in Georgia under the Laws on Health Care and the Protection of Experimental Animals.

### Animals and treatments

Experiments were carried out on adult (150-170 g) and adolescent (7, 10, 21, 28 days) white rats, adult pigeons and mice. Animals were housed under controlled conditions at a temperature of 25 ± 2˚C, relative humidity of 60 ± 10%, with room air changes 12-18 times/hour, and a dark/light ratio=14/10. During the experiment, they were provided with unrestricted access to water and food. The animals were divided into two groups: the control group, in which the animals were injected with 100 μl 0.9% saline; and the test group, in which the animals were injected with rat brain TPC (200 γ) intraperitoneally (as described previously) (17). The animals in both groups were decapitated under ether anesthesia three hours later. Brain, kidney and liver tissues were fixed in 4% paraformaldehyde (Sigma, USA) solution prepared in 0.1 M phosphate buffered saline pH=7.4 (Sigma, USA). The samples were embedded in paraffin, sectioned using a microtome and stained using standard [Hematoxylin and eozine (H&E)] and immunohistochemical protocols. Tissue samples were studied under the light microscope (Zeiss Primo Star, Germany).

### Isolation of brain thermostable protein complex

TPC was obtained by alcohol extraction from a normal adult rat brain as previously described (18). The rat brain tissue was rinsed with 0.9% saline and crushed. Aqueous homogenates were prepared in a tissue/distilled cold water ratio of 1: 8. Then 96% ethanol were added twice to homogenates to obtain a protein fraction of 81% ethanol After centrifugation precipitate were solved in distilled water and boiled in a water bath (100˚C) for 20 minutes and centrifuged (600 g, 15 minutes). The supernatant was frozen and dried in a lyophilizer. As a result, a white powder residue of the TPC, soluble in water, was obtained. The protein samples were stored at 4˚C. The method of Lowry et al. (19) was used for measuring protein concentrations.

### Determination of mitotic index

1 mg/kg of colchicine (Sigma, USA) was injected into the animals of both the control and the test groups for the determination of the colchicine mitotic index per 1000 cells (‰).

### Immunohistochemistry

Blocking of endogenous peroxidase with 3% hydrogen peroxide was performed on dewaxed and rehydrated slides for 10 minutes. Heat induced epitope retrieval in citrate buffer pH=6 (Abcam, UK) was used for 3 minutes (in microwave oven) to recover the Ki67 epitope. Tris-EDTA buffer pH=9.0 (Abcam, UK) was used as the antigen retrieval procedure for GAD65/67. Incubation with primary antibodies Ki67-clone SP6 (Abcam, UK, dilution 1: 50) and GAD65/67 (Sigma, USA, dilution 1: 1000), for two hours proceeded with the application of biotinized secondary antibodies (Sigma, USA, dilution 1: 400). Immunoreactivity was visualized using extravidin peroxidase (Sigma, USA, dilution 1: 200) and 3, 3’-diaminobenzidine (SIGMAFAST™ DAB, Sigma, USA) as chromogen. For each sample 5000 cells were counted and the number of Ki67 and GAD65/67 positive cells per 1000 cells (‰) was determined. Images were taken with a Zeiss Primo Star microscope.

### Evaluation of transcriptional activity

The transcriptional activity of the tissues was evaluated by the test-system of the isolated nuclei (20). Nuclear fractions were incubated in 0.2 ml solution of Tris-HCL buffer (pH=8.3, Sigma, USA)-50 μm; MgCL2 (Sigma, USA)-7.5 μm; ATP, CTP, GTP-each 0.05 μm; [^14^C]-UTP (“UVVV”, Czechoslovakia, specific activity 4.3 GBq/ mM), 0.001 μm. Each nuclear sample contained 100- μg DNA. Transcriptional activity was determined by the intensity of [^14^C] -UTP uptake in scintillator detector SL30 (Intertechnique, France).

### Statistical analysis

Data are expressed as mean SD. Students’ t test was used for comparison among the different groups. P<0.05 was considered statistically significant.

## Results

### The influence of rat brain thermostable protein complex on the transcriptional activity of various tissues

**Fig.1:**
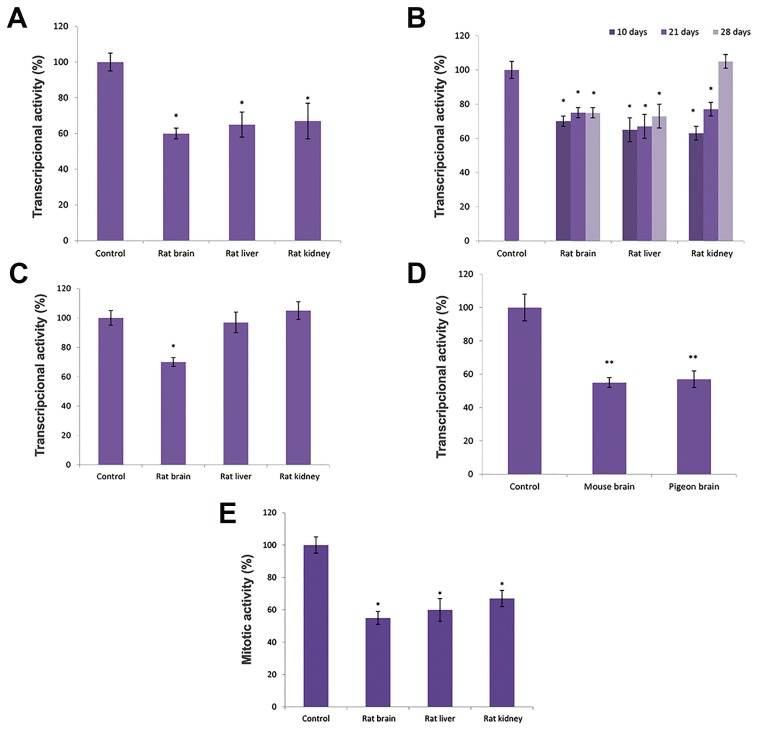
The Influence of rat brain thermostable protein complex (TPC) on the transcriptional activity of various tissues of different animals. A. Changes in transcriptional activity in various tissues of the adolescent (7-day-old) rat, B. Changes in transcriptional activity in various tissues of different age rats, C. Changes in transcriptional activity in various tissues of adult rats, D. Changes in transcriptional activity of isolated nuclei from the mice and pigeons brain, and E. Influence of rat brain TPC on the mitotic activity of various organs in adolescent rats. *; P<0.05 comparison to control in each group and **; P<0.01 comparison to control in each animal group.

The transcriptional activity of nuclei isolated from the tissues of adolescent rats (7 days old) was decreased, on average by 35%, by the presence of rat brain TPC (Fig .1A). The same effect was also seen in nuclei isolated from the tissues of 10 and 21 day-old rats. Transcriptional activity was inhibited by 35% by rat brain TPC in all three organs; brain, kidney and liver. Different results were obtained in the case of 28 day-old rats. TPC from rat brain had no effect on the transcriptional activity of nuclei isolated from the kidney (Fig .1B). The next stage of the research, which used the tissues of adult rats, established that the inhibitory effect of brain TPC occurred only in homotypic cells (Fig .1C).

### The species specificity of rat brain thermostable protein complex

#### The impact of brain thermostable protein complex on the transcriptional activity of brain cells of various species

The brain tissues of adult white mice and pigeons were used to examine the species specificity of rat brain TPC. It was found that rat brain TPC decreased RNA synthesis by an average of 35-50% in nuclei isolated from the brain cells of both species (Fig .1D).

### The effect of thermostable protein complex on mitotic activity

The goal of the next stage of the investigation was to determine whether the inhibition of transcription would affect important processes, such as cell proliferation, in adolescent rats. We therefore studied the effect of rat brain TPC on the mitotic activity of various organs (brain, kidney, and liver) in adolescent (7-day-old) rats. It was found out that a single intraperitoneal injection of rat brain TPC leads to an average decrease of 30-40% in the mitotic index of both homotypic and heterotypic cells compared to controls (Fig .1E).

### The influence of thermostable protein complex on the proliferative activity of cells in dentate gyrus

**Fig.2:**
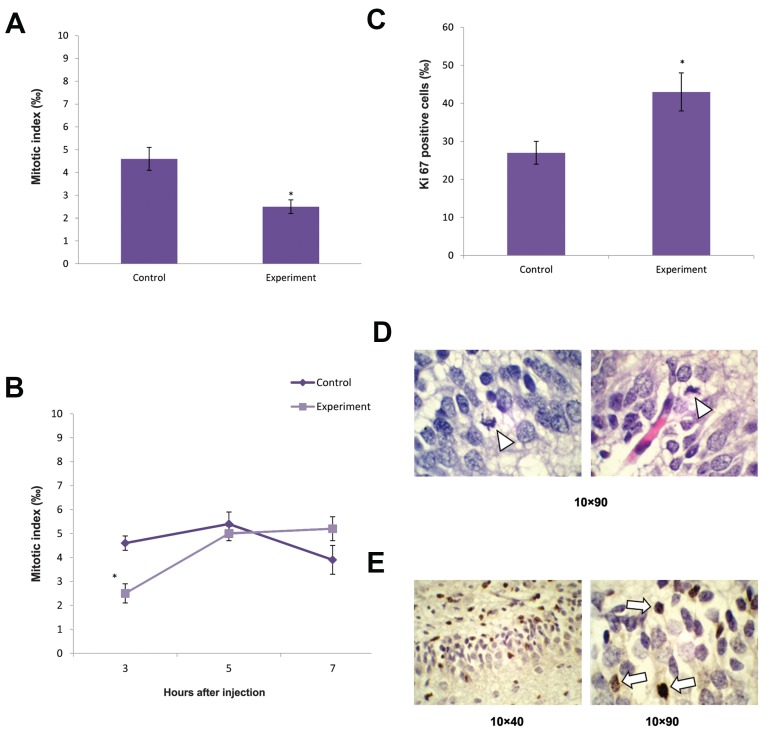
The influence of rat brain thermostable protein complex (TPC) on the proliferative activity of dentate gyrus cells of adolescent rats. A. Changes in mitotic activity in the dentate gyrus, B. The dynamic of changes in dentate gyrus mitotic activity, C. Changes in numbers of Ki 67 positive cells in the dentate gyrus, D. Mitotic figures (arrowheads) in the dentate gyrus (X90, H&E), and E. Ki 67 positive cells (arrows) in the dentate gyrus (X40 and X90). *; P<0.05 comparison to control.

The influence of a single injection of rat brain TPC on cell proliferation in the dentate gyrus was evaluated after 3, 5 and 7 hours. These investigations revealed a decrease in the mitotic activity of the progenitor cells of about 35% three hours after TPC injection (Fig .2A). The colchicine mitotic index of these cells did not change when evaluated 5 and 7 hours after the injection. In both cases the mitotic index (3.4-3.6 ‰) does not exceed the corresponding values for the control group (Fig .2B). Changes in cell proliferation intensity in the hippocampus were also estimated using the antibody against proliferation marker-Ki67. This part of the study showed that as the mitotic index in the hippocampus decreased, the number of Ki67 positive cells increased by 36% (Fig .2C).

### The effect of thermostable protein complex on the quantity of GAD65/67 positive cells in the dentate gyrus

**Fig.3:**
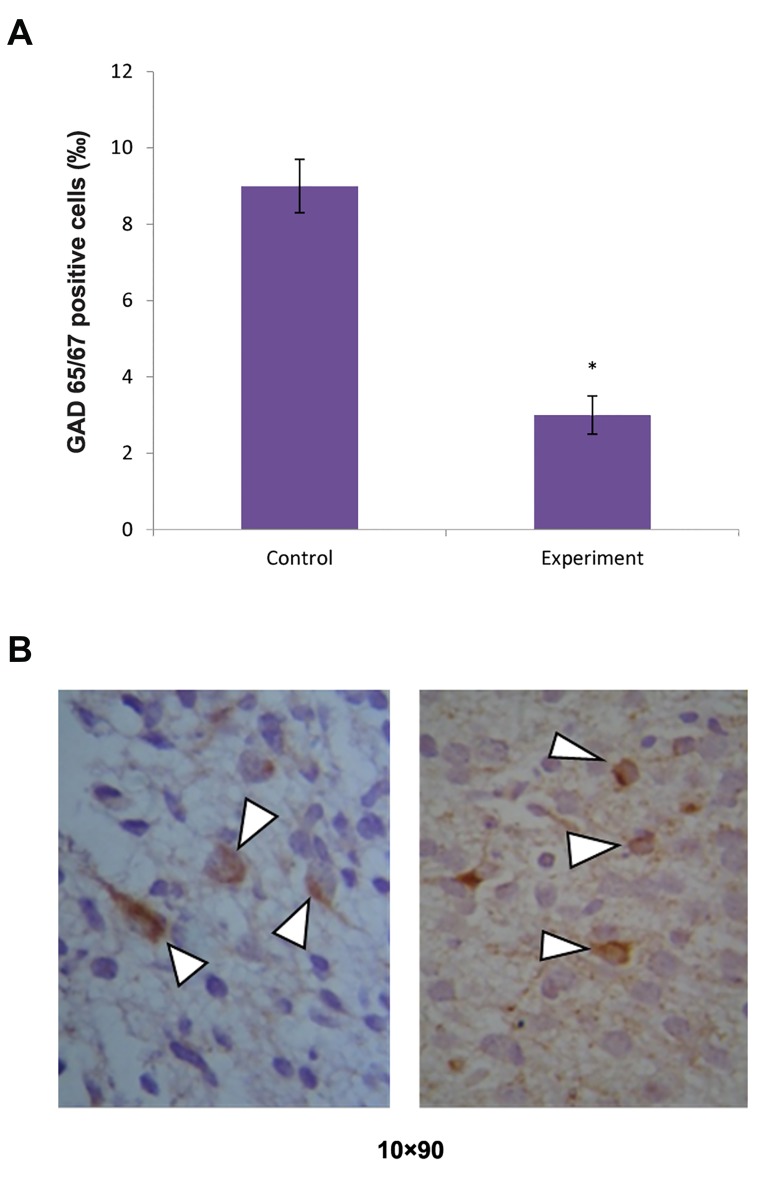
The influence of rat brain thermostable protein complex (TPC) on the quantity of GAD65/67 positive cells in the dentate gyrus of adolescent rats. A. Changes in the number of GAD65/67 positive cells and B. GAD65/67 positive cells (arrowheads, X90). *; P<0.05 comparison to control.

The influence of rat brain TPC on the number of GAD65/67 positive cells in the rat hippocampus was studied. After 3 hours it was found that injections of rat brain TPC decreased the number of GAD65/67 positive cells in the hippocampus by approximately 3-fold compared to the control group (Fig .3A).

## Discussion

Intracellular and extracellular factors that regulate cell proliferation promote cell cycle initiation or the blocking and exit of cells from the cell cycle (21). In some cases, factors involved in the signaling pathways are multifunctional substances. It has been shown that fibroblast growth factors (e.g. FGF 2) have several and often controversial functions and that growth factors affect different phases of the cell cycle (22, 23). The participation of transmembrane proteins, integrins, in the regulation of cell proliferation has been described (24). All these factors are important for embryonic development, as well as in adults to maintain cellular balance. Their action is carried out by different mechanisms, one of which is gene activation via intracellular cascading reactions. The passage of cells through the cell cycle phases is controlled by the products expressed by these genes-the so-called positive and negative factors (25). The loss or the weakening of this control mechanism may be the cause of many serious diseases. Therefore, firstly, we examined the effects of rat brain TPC on transcriptional activity of the tissues. Our studies showed that the TPC decreases the intensity of RNA synthesis in the cell nuclei of adolescent (7-day-old) rat brains, as well as in the adult rat brain.

The inhibition of transcription in turn should have effects on mitosis, and indeed our results show a decrease in the mitotic index 3 hours after the injection of rat brain TPC. An analogous effect has already been described for the protein complexes from rat heart and kidney cells (5, 6). Only specific factors have the ability to regulate the proliferation of certain cells (26). Taking the fact that rat brain TPC has an inhibitory effect on the mitotic index of different organs (brain, liver, and kidney) in adolescent (7-day-old) animals, we can assume that we are dealing with a non-specific action. Studying the impact of rat brain TPC on an adult rat tissue reveals that TPC has no inhibitory effect on the transcriptional activity of liver and kidney cells in the adult animal. Hence TPC from rat brain is tissue specific, but this feature is not revealed in the early stages of postnatal development.

So, naturally, the question arises - when does the action of TPC on heterotypic cells become limited?

To answer this question, we used several tissues from rats at different stages of postnatal development over the first month after birth. Our results show that transcriptional activity is suppressed by TPC at the same rate in brain, liver and kidney cells during the first three weeks after birth. A different picture is observed in the case of 4-week old rats with regard to liver and kidney cells. While the transcriptional activity is still inhibited in hepatocytes the intensity of RNA synthesis remains unchanged in kidney cells. This result indicates that the effect of TPC in different cell types is restricted to certain stages of development. At the early stage of development in rats, as is well known, the growth of various organs through cell proliferation ends at different times. For example, proliferation of heart tissue is terminated at the end of the third week after birth, but in liver tissue this process continues until the 6th week (27, 28). This explains why we see inhibition of RNA synthesis by rat brain TPC in liver cells of 4-week old rats in the present study.

According to the literature, growth factors are not characterized by species specificity (29). To study species specificity we used brain tissue from an adult mouse and a pigeon. Our results showed that rat brain TPC is not characterized by species specificity, similar to TPC from rat heart and kidney (5, 17). The results obtained from various adolescent tissues, as well as from adult animals enable us to conclude that TPC is not species-specific, but expresses tissue specificity with regard to terminally differentiated cells.

As the brain is characterized by plasticity, due to the presence of progenitor cells (11), and the dentate gyrus of the hippocampus is the main source of progenitor cells for neurons as well as glial cells (10), in the next stage of the research we studied the impact of rat brain TPC on the proliferative activity of cells in the dentate gyrus of adolescent rats. As the results show, rat brain TPC has the ability to inhibit the mitotic activity of the progenitor cells. In a further series of experiments, we tried to find out whether the process is reversible or not, specifically whether the mitotic index is decreased by delaying cell movement into the mitosis phase or by cell death. For this purpose, we increased the duration of the exposure to rat brain TPC by two hours (the standard duration of an experiment was 3 hours). The observed increase in the mitotic index in the dentate gyrus, 5 hours after injection of rat brain TPC could only be explained by the unblocking of G2-to-M phase transition in cell cycle. Thus, the decrease in mitotic activity associated with rat brain TPC in the first 3 hours occurs as a result of a temporary delay of cells in the G2-phase.

After using the antibody against proliferation marker Ki67 a different picture was seen. The number of cells in the cell cycle increased 3 hours after the injection of rat brain TPC. The increase of Ki67 positive cells associated with the observed reduction in the mitotic index could occur in two ways. Firstly, the increasing of number of Ki67 positive cells (cells in the cell cycle) in the dentate gyrus of the experimental animals, can be due to the number of cells delayed in the G2 phase. Secondly, in the tissue programmed to growth and proliferation, the delaying of cells in the cell cycle by the rat brain TPC can cause the entrance of a new population of precursor cells into the cell cycle.

The ability to accelerate the entrance of new cell populations into the cell cycle is confirmed by the reduced number of GAD65/67 positive cells observed. Decreasing of this enzyme expression in animals in the experimental group indicates a reduction of the transformation of glutamate into gamma aminobutyric acid. According to the literature, glutamate causes strong, progressive activation of the ERK and JNK/ SAPK MAPK cascades (30). Our immunohistochemical analysis indicates that the number of cells in the cell cycle increases through the glutamate activation of these cascades.

## Conclusion

From our results it follows that rat brain TPC causes the reversible inhibition of cell proliferation through the inhibition of transcription. TPC is not characterized by species specificity, while tissue specificity appears to be limited to terminally differentiated cells. In the early stage of postnatal development, the inhibitory effect of rat brain TPC causes an increase in the number of cells in the cell cycle that is achieved by switching on reserve mechanisms in tissues programmed to proliferation.

## References

[1] Tarsounas M, Pearlman RE, Gasser PJ, Park MS, Moens PB (1997). Protein-protein interactions in the synaptonemal complex. Mol Biol Cell.

[2] Kuzmanov U, Andrew E (2013). Protein-protein interaction networks: probing disease mechanisms using model systems. Genome Med.

[3] Toepke MW, Impellitteri NA, Lan Levengood SK, Boeldt DS, Bird IM, Murphy WL (2012). Regulating specific growth factor signaling using immobilized branched ligands. Adv Healthc Mater.

[4] Lindskog C, Korsgren O, Pontén F, Eriksson JW, Johansson L, Danielsson A (2012). Novel pancreatic beta cell-specific proteins: antibody- based proteomics for identification of new biomarker candidates. J Proteomics.

[5] Giorgobiani N, Rusishvili L, Dzidziguri D, Salakaia T, Tumanishvili G (2002). The investigation of specie and tissue specificity of cardiomyocytes growth inhibiting factor. Proc Georgian Acad Sci Biol Ser A.

[6] Rukhadze MD, Dzidziguri DV, Giorgobiani NM, Kerkenjia SM (2005). The study of growth inhibitive protein factor by various mode of HPLC and estimation of its binding with drugs. Biomed Chromatogr.

[7] Dzidziguri D, Aslamazishvili T, Chkhobadze M, Khorava P, Chigogidze T, Managadze L (2004). The influence of white rat protein factor on transcriptional activity of normal and transformed cells. Proc Georgian Acad Sci Biol Ser B.

[8] Eriksson PS, Perfilieva E, Björk-Eriksson T, Alborn AM, Nordborg C, Peterson DA (1998). Neurogenesis in the adult human hippocampus. Nat Med.

[9] Johnson PF (2005). Molecular stop signs: regulation of cell-cycle arrest by C/EBP transcription factors. J Cell Sci.

[10] Fagel DM, Ganat Y, Silbereis J, Ebbitt T, Steward W, Zhang H (2006). Cortical neurogenesis enhanced by a chronic perinatal hypoxia. Exp Neurol.

[11] Leuner B, Gould E (2010). Structural plasticity and hippocampal function. Annu Rev Psychol.

[12] Grote HE, Hannan AJ (2007). Regulators of adult neurogenesis in the healthy and diseased brain. Clin Exp Pharmacol Physiol.

[13] Schänzer A, Wachs FP, Wilhelm D, Acker T, Cooper-Kuhn C, Beck H (2004). Direct stimulation of adult neural stem cells in vitro and neurogenesis in vivo by vascular endothelial growth factor. Brain Pathol.

[14] Seri S, Cerquiglini A, Harding GF (2006). Visually induced syncope: a nonepileptic manifestation of visual sensitivity?. Neurology.

[15] Aberg MA, Aberg ND, Hedbäcker H, Oscarsson J, Eriksson PS (2000). Peripheral infusion of IGF-I selectively induces neurogenesis in the adult rat hippocampus. J Neurosci.

[16] Jin K, LaFevre-Bernt M, Sun Y, Chen S, Gafni J, Crippen D (2005). FGF-2 promotes neurogenesis and neuroprotection and prolongs survival in a transgenic mouse model of Huntingtons’ disease. Proc Natl Acad Sci USA.

[17] Dzidziguri DV, Chigogidze TG, Managadze LG, Aslamazishvili TT, Kerkenjia SM (2007). Kidney protein complexes that inhibit gene expression in the nuclei of homotypic cells. Georgian Med New.

[18] Giorgobiani N, Dzidziguri D, Rukhadze M, Rusishvili L, Tumanishvili G (2005). Possible role of endogenous growth inhibitors in regeneration of organs: searching for new approaches. Cell Biol Int.

[19] Lowry OH, Rosebrough NJ, Farr AL, Randall RJ (1951). Protein measurement with the folin phenol reagent. J Biol Chem.

[20] Dzidziguri DV, Chelidze PV, Zarandia MA, Cherkezia EC, Tumanishvili GD (1994). Transcriptional activity and ultrastructure of morphologically different types of nucleoli isolated from hepatocytes of normal and hepatectomized rats. Epithelial Cell Biol.

[21] Francis SM, Bergsied J, Isaac CE, Coschi CH, Martens AL, Hojilla CV (2009). A functional connection between pRB and transforming growth factor beta in growth inhibition and mammary gland development. Mol Cell Biol.

[22] Balažs A, Blažsek I (1979). Control of cell proliferation by endogeneous inhibitors.

[23] Salotti J, Dias MH, Koga MM, Armelin HA (2013). Fibroblast growth factor 2 causes G2/M cell cycle arrest in ras-driven tumor cells through a Src-dependent pathway. PLoS One.

[24] Bill HM, Knudsen B, Moores SL, Muthuswamy SK, Rao VR, Brugge JS (2004). Epidermal growth factor receptor-dependent regulation of integrin-mediated signaling and cell cycle entry in epithelial cells. Mol Cell Biol.

[25] Marqués M, Kumar A, Cortés I, Gonzalez-García A, Hernández C, Moreno-Ortiz MC (2008). Phosphoinositide 3-kinases p110alpha and p110beta regulate cell cycle entry, exhibiting distinct activation kinetics in G1 phase. Mol Cell Biol.

[26] Sweeney C, Fambrough D, Huard C, Diamonti AJ, Lander ES, Cantley LC (2001). Growth factor-specific signaling pathway stimulation and gene expression mediated by ErbB receptors. J Biol Chem.

[27] Sidorova VF (1969). Postnatal growth and renewal of internal organs in vertebrates.

[28] Romanova LK (1984). Regulation of renewal processes.

[29] Takahashi T, Tanaka M, Inazawa J, Abe T, Suda T, Nagata S (1994). Human Fas ligand: gene structure, chromosomal location and species specificity. Int Immunol.

[30] Vanhoutte P, Barnier JV, Guibert B, Pagès C, Besson MJ, Hipskind RA (1999). Glutamate induces phosphorylation of Elk-1 and CREB, along with c-fos activation, via an extracellular signal-regulated kinase- dependent pathway in brain slices. Mol Cell Biol.

